# Supplementary material for: Secular Trends in Ablation Therapy for Graves’ Disease: An Analysis of a 15-Year Experience at a Tertiary Hospital in South Korea
Source: J Clin Med. 2021 Apr 12;10(8):1629. doi: 10.3390/jcm10081629 (PMC8069724; doi:10.3390/jcm10081629)

Supplementary Table S1. Adverse effects of ATD requiring the ablation therapy.

	Total	Methimazole	PTU	*p*
Total number [a]	10,986	9,939	2,333	
Number of adverse events, n (%) [b]	154 (1.4)	110 (1.1)	66 (2.8)	<0.01
Toxic hepatitis, n (%)	69 (0.6)	47 (0.5)	26 (1.1)	<0.01
Agranulocytosis, n (%)	45 (0.4)	32 (0.3)	13 (0.6)	0.08
Urticaria/skin rash, n (%)	34 (0.3)	30 (0.3)	27 (1.2)	<0.01

ATD, anti-thyroid drug; PTU, propylthiouracil.
[a] Among total patients, 1,286 patients used both ATDs.
[b] 22 patients had adverse effects to both ATDs.

Supplementary Figure S1. Remission rate for RAI therapy and surgery over time.

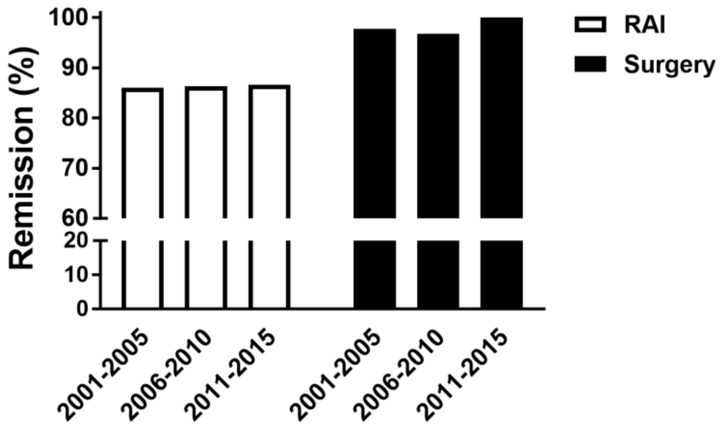

Supplement: Supplementary file 1 [file jcm-10-01629-s001.pdf]
